# Identification and Phylogenetic Classification of *Fasciola* species Isolated from Sheep and Cattle by PCR-RFLP in Zabol, in Sistan and Baluchistan Province, Southeast Iran

**Published:** 2019-05

**Authors:** Sedighe MIR, Mansour DABIRZADEH, Mohammad Bagher ROKNI, Mojgan ARYAEIPOUR, Mahdi KHOSHSIMA SHAHRAKI, Hakim AZIZI

**Affiliations:** 1. Student Research Committee, Zabol University of Medical Sciences, Zabol, Iran; 2. Department of Medical Parasitology, School of Medicine, Zabol University of Medical Sciences, Zabol, Iran; 3. Department of Medical Parasitology and Mycology, School of Public Health, Tehran University of Medical Sciences, Tehran, Iran; 4. Center for Research of Endemic Parasites of Iran (CREPI), Tehran University of Medical Sciences, Tehran, Iran

**Keywords:** ITS1, PCR-RFLP, Genotyping, *Fasciola hepatica*, *Fasciola gigantica*, Iran

## Abstract

**Background:**

The detection of *Fasciola* species in various geographical regions is essential for health policymaking. Here, we aimed to identify livestock (cattle and sheep) related *Fasciola* genotypes by restriction fragment length polymorphism PCR.

**Methods:**

Seventy adult *Fasciola* flukes were collected from 70 infected livers of 35 cattle and 35 sheep slaughtered in Zabol abattoir, south–east Iran (Jan–Jul 2017). *Fasciola* species were determined based on molecular features. For molecular detection, *Fasciola* ITS1 region was amplified and sequenced. A 700 bp fragment was amplified. These were digested with RasΙ enzyme. *F. hepatica* specific fragments were 47, 59, 68, 104, and 370, while those related to *F. gigantica* had 45, 55, 170, 370.

**Results:**

The two main species of *F. hepatica* and *F. gigantica* are responsible for fasciolosis in sheep and cattle in our region. From 35 *Fasciola* isolated from cattle, 3 and 32 were *F. hepatica* and *F. giagantica* respectively. From 35 *Fasciola* isolated from sheep, 4 were *F. hepatica* and 31 were *F. gigantica.*

**Conclusion:**

All Seventy *Fasciola* samples from two different hosts (cattle and sheep) were identified as either *F. hepatica* or *F. gigantica* by PCR-RFLP. Genotypic variability of *Fasciola* species was high in our region. It is recommended to assess molecular variation of *Fasciola* isolates in other host livestock.

## Introduction

Fascioliasis is a trematode flatworm infection caused by ***Fasciola hepatica*** or ***F. gigantica*** in livestock and humans (1). These two species represent high variability across the world. In particular, despite that only *F. hepatica* has been mainly encountered in Europe, the US, and Australia, both genus have been reported from Asia (including Iran) and Africa (2, 3). Fasciolosis has been considered as an important disease in both medicine and veterinary (1).

After an increasing trend of human fasciolosis in recent decades, the disease has been focused as a major health problem by the WHO. Fasciolosis has been categorized as an important food-born parasitic infection by WHO (4).

Fasciolosis has been reported from more than 60 countries around the world. Until now, substantial rates of 2.4 to 17 million people are infected, and 91 million at risk of the infection have been estimated (1, 5). Iran is one of the endemic regions for fasciolosis. The number of individuals at risk of fasciolosis has been estimated at 6 million in Iran (6). Two great fasciolosis epidemics in northern Iran during 1989 and 1999 turned the epidemiologic picture of the infection in Iran (7–11).

These two species differ from each other regarding transmission, epidemiologic, and phylogenic properties. Each of these species is hosted by different species of **Lymnaea** snails (5). Despite the fact that the two parasitic species can be differentiated based on morphogenic characteristics, the exact identification may be hindered due to multiple intermediate hybrid parasitic forms (12, 13).

Considering the health and economic burden of fasciolosis, identification of *F. hepatica* and *F. gigantica* species is deemed essential for implementing appropriate preventive measure (1). This is also essential to overcome shortcomings of phenotypic approach, and it is necessary to recruit genotypic methods to accurately and rapidly identify *Fasciola* species. This is also needed to effectively manage the infection and provide appropriate epidemiologic evidence regarding the distribution of the infection (14–17).

Ribosomal DNA (rDNA) are useful markers in genomic studies as these are sequences enclosing both variable and constant segments, and also are available in large copy numbers (3,12). First and second internal transcribed spacers (ITS-1 and ITS-2) of rDNA can present useful markers for identifying *Fasciola* species (12, 15, 16). Other molecular markers used are mitochondrial DNA sequences (mtDNA). These are in particular appropriate for discerning closely related organisms at both species and subspecies levels (18, 19). In the recent years, several PCR based techniques have been developed (i.e. PCR-linked restriction fragment length polymorphism (PCR-RFLP), PCR-linked single-strand conformation polymorphism (PCR-SSCP) and specific PCR assays) for accurate differentiating of *Fasciola* species (20–22).

Despite detection of fasciolosis infection in abattoirs of Zabol City, molecular studies on these parasites are limited in this region (23). As there was no data on *Fasciola* species infected livestock in Zabol, we aimed to characterize these species in infected sheep and cattle slaughtered in Zabol abattoirs using molecular detection of ITS1 region. We then aligned these sequences with recorded sequences of *Fasciola* in the GenBank database.

## Materials and Methods

### Parasites

This study was performed in Zabol, located in north of Sistan and Baluchistan Province, south-east of Iran (Jan–Jul 2017). After abstaining primary agreement of abattoir manager, 70 infected livers from cattle and sheep were collected. Seventy flukes were isolated from 35 infected cattle and 35 infected sheep. The flukes were isolated and washed with PBS buffer (37 °C). A unique code was assigned to each parasite considering its species and the host. The flukes were preserved in ethanol 40% and room temperature until DNA extraction.

The study was approved by Ethics Committee of the university.

### DNA extraction

Ten mg of dorsal parts of the flukes were removed using a specific blade. The samples were left in room temperature until the ethanol was evaporated. After that, the samples were rinsed in distilled water for 6 times and then were homogenized with sonication. DNA was extracted using DNG-^TM^-plus. The DNA was preserved in −20 until use.

### Primer design

A 700 bp segment of ITS1 region was amplified. Forward and reverse primer sequences were those designed in our previous study and were as 5′-ACCGGTGCTGAGAAGACG-3′ and 5′-CGACGTACGTGCAGTCCA-3 respectively. These were synthesized by Bioneer (South-Korea) (17).

### PCR

PCR reaction was performed in a net volume of 15 μL. The mixture comprised 1.5 μL DNA template, 5 μL distilled water, 1 μL of 10 pmol forward and reverse primers, and 7.5 μL Taq polymerase master mix. The PCR reaction was run in thermocycler (Labnet, USA). Initial denaturation phase was accomplished at 95 °C for 5 min. Thirty PCR cycles comprising 30 sec of denaturation at 94 °C, 30 sec of annealing at 60 °C, and 30 sec of extension at 72 °C were done. In the end, the mixture was remained at 72 °C for 5 min to complete the reaction. The amplicons were chilled on ice and then were visualized by agarose gel electrophoresis.

### PCR-RFLP

Initially, we applied the Web cutter software for finding restriction points on *F. hepatica* and *F. gaigantica* sequences to find appropriate restriction enzyme. Based on this, RsaΙ enzyme was selected. This enzyme detects and excises the “GTAC” sequence. To perform RFLP, a mixture containing 5 μL PCR amplicon, 2.5 μL restriction enzyme buffer, and 5 μL of diluted restriction enzyme was prepared. The net volume was reached to 25 μL by adding DNase free distilled water. The reaction mixtures were incubated at 37 °C for 7 h. The digested DNA segment was checked by 3% gel agarose electrophoresis using ethidium bromide.

### DNA sequencing and phylogenic analysis

PCR products of ITS1 region of 15 isolates obtained from either cattle or sheep were sequenced using PCR amplicons and forward and reverse primers. The sequences were then aligned according to the sequences available at the GenBank.

## Results

Genomic DNA was extracted from 70 *Fasciola* isolates, and 15 (eight from cattle and seven from sheep) were sequenced. A 700 bp segment was amplified. According to the length of the amplicon, there was no difference between isolates of cattle and sheep. These were therefore digested with a restriction enzyme to differentiate the isolates.

### PCR-RFLP Analysis

**Fig. 1:**
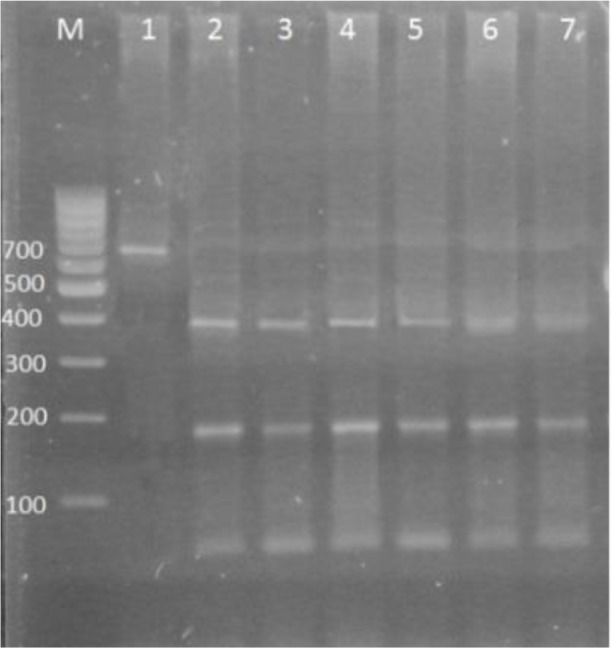
(*F. gigantica*) Gel electrophoresis, showing PCR-RFLP results after digestion with the restriction enzyme RsaΙ on samples. Lane 2,3 and 4 *F. gigantica* isolated from sheep; Lanes 5,6 and 7 *F. gigantica* isolated from cattle; Lane 1 negative control; M: molecular weight marker (100bp)

**Fig. 2:**
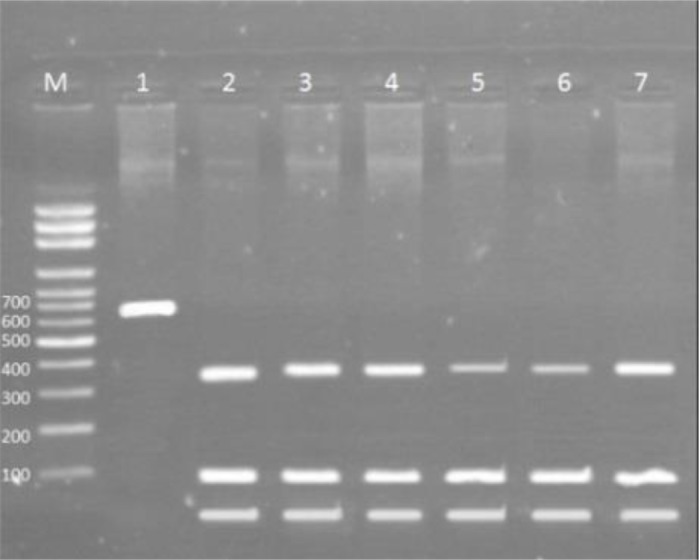
(*F. hepatica*) Gel electrophoresis showing PCR-RFLP results after digestion with the restriction enzyme RsaΙ on samples. Lane 2,3 and 4 *F. hepatica* isolated from sheep; Lanes 5,6 and 7 *F. hepatica* isolated from cattle; Lane 1 negative control; M: molecular weight marker (100bp)

RFLP pattern for *F. hepatica* showed four restriction sites and 47, 59, 68, 104, and 370 bp segments. The pattern for *F. gigantica* showed three restriction sites and 45, 55, 170, 370 bp fragments. From 35 *Fasciola* isolated from cattle, 3 (8.5%) and 32 (91.5%) were *F. hepatica* and *F. giagantica* respectively. From 35 *Fasciola* isolated from sheep, 4 (11.4%) were *F. hepatica* and 31 (88.6%) were *F. gigantica* (Figs. 1, 2).

**Fig. 3:**
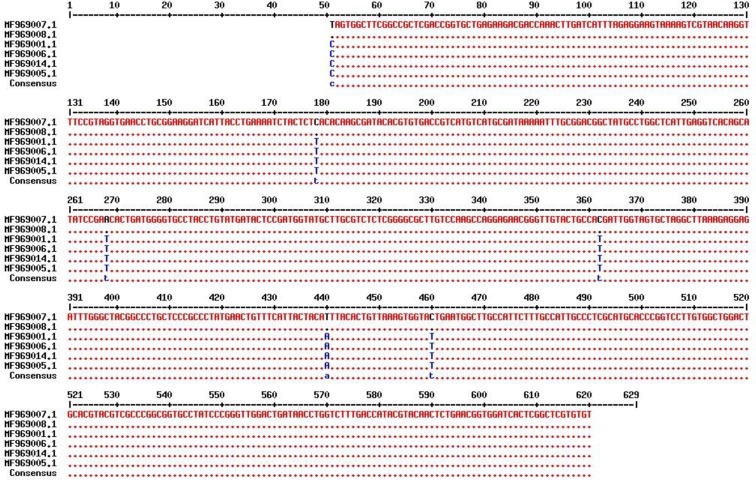
Sequence alignment of the ITS1, region from *Fasciola hepatica* (MF969007& MF969008) and *Fasciola gigantic* (MF969001, MF969005, MF969006& MF969014) of Zabol, Iran

**Fig. 4:**
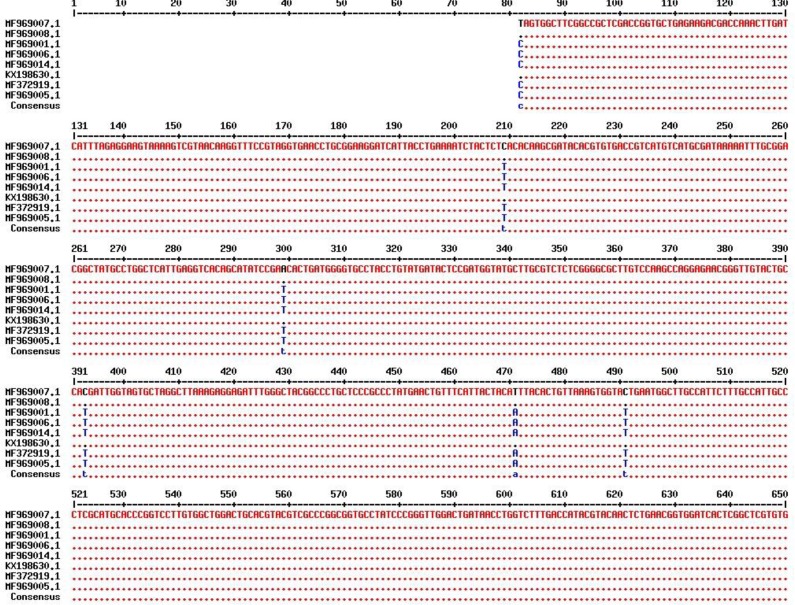
Alignment of ITS1 sequence of *F. hepatica* (KX198630.1) and *F. gigantica* (MF372919.1) deposited in GenBank with *F. hepatica* (MF969007& MF969008) and *F. gigantica* (MF969001, MF969005, MF969006& MF969014) of Zabol, Iran

The ITS1 700 bp region of the flukes was aligned against the sequences available at the GenBank using Multalin software (Figs. 3, 4). This showed variable nucleotides at 51, 178, 268, 362, 440, 460 ancestry positions (Table 1).

**Table 1:** Position of variable bases and differences in sequences of ITS1 region of *Fasciola* spp

***Position sample***	***Haplotype***	***Accession Number***	***ITS1***					
			***51***	***178***	***268***	***362***	***440***	***460***
*F. hepatica*	(H1)	MF969008	T	C	A	C	T	C
*F. hepatica*	(H2)	MF969007	T	C	A	C	T	C
*F. gigantica*	(G1)	MF969006	C	T	T	T	A	T
*F. gigantica*	(G2)	MF969005	C	T	T	T	A	T
*F. gigantica*	(G3)	MF969014	C	T	T	T	A	T
*F. gigantica*	(G4)	MF969001	C	T	T	T	A	T

We also analyzed and deposited some sequences of *F. gigantica* and *F. hepatica* (Table 2)*.* The similarity of sequences detected in present study with those present at the GenBank showed 98%–100% homology.

**Table 2:** Profile of *Fasciola* spp. used in this study

***Specimen code***	***species***	***Haplotyp***	***Host***	***Sequence analysis***	***ITS1 types Accession no.***	***PCR-RFLP Analysis***
FhB9	*F.hepatica*	H2	Cattle	*F.hepatica*	MF969007	*F.hepatica*
FhS1	*F.hepatica*	H1	Sheep	*F.hepatica*	MF969008	*F.hepatica*
FhS2	*F.hepatica*	H2	Sheep	*F.hepatica*	MF969009	*F.hepatica*
FhS8	*F.hepatica*	H1	Sheep	*F.hepatica*	MF969010	*F.hepatica*
FGB12	*F.gigantica*	G4	Cattle	*F.gigantica*	MF969001	*F.gigantica*
FGB42	*F.gigantica*	G4	Cattle	*F.gigantica*	MF969004	*F.gigantica*
FGS4	*F.gigantica*	G2	Sheep	*F.gigantica*	MF969005	*F.gigantica*
FGS5	*F.gigantica*	G1	Sheep	*F.gigantica*	MF969006	*F.gigantica*
FGS12	*F.gigantica*	G4	Sheep	*F.gigantica*	MF969011	*F.gigantica*
FgB1	*F.gigantica*	G4	Cattle	*F.gigantica*	MF969012	*F.gigantica*
FgB2	*F.gigantica*	G1	Cattle	*F.gigantica*	MF969013	*F.gigantica*
FgB3	*F.gigantica*	G3	Cattle	*F.gigantica*	MF969014	*F.gigantica*

### Phylogenic tree

**Fig. 5:**
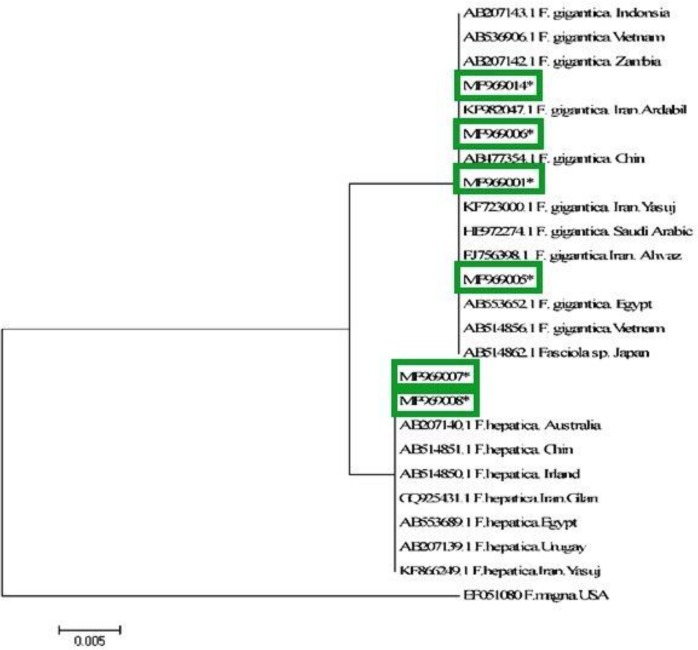
Phylogenetic relationship of ITS1 sequences of isolates of *F. hepatica* and *F. gigantic* from Iran using Maximum likelihood method. *Fasciola magna* (AN: EF051080) was used as the out-group.

The phylogenic tree of ITS1 region showed dispersion of *F. hepatica* and *F. gigantica* flukes in Zabol region. These genotypes had the ability to infect animals and probably humans (Fig. 5).

## Discussion

Regarding availability, and being cheap, morphometric identification can be helpful in initial discrimination of *Fasciola* species. However, the precise identification and segregation of species, particularly in case of intermediate forms, necessitates molecular evaluations (24, 25).

In present study, we recruited the relatively fast and reliable method of PCR-RFLP to detect and discriminate *Fasciola* species collected from cattle and sheep in Zabol City. A 700 bp segment (encompassing ITS1, and 18S rRNA) was amplified. The fragment was then digested with RsaΙ enzyme. This procedure created specific and differentiable patterns for *F. hepatica* and *F. gigantica* species. We did not find any intraspecific variations in our study.

Our results are in line with the results that used RsaΙ enzyme to digest ITS1 and found an intermediate species in addition to *F. hepatica* and *F. gigantica* (26). In other studies, ITS1 enzyme was exploited to differentiate the two *Fasciola* species in the North-West and the South-East of Iran, respectively. No intermediate species were reported by the two recent studies (16, 27).

In a study, samples isolated from cattle were evaluated in Iranshhar and Zabol of Sistan and Baluchistan. *F. hepatica* did not show any genetic variation (because of merely detection of cattle isolates), while high genetic variability found in *F. gigantica* (28).

Multiple studies have been performed to identify *Fasciola* species using PCR-RFLP in Iran. The majority of these studies were successful in isolating the two *Fasciola* species. Nevertheless, Karimi et al. failed to differentiate these species using BfrΙ, while they successfully discriminated the species using DraΙ enzyme (24). BamHΙ and PagΙ enzymes had no effects on ITS2 region of *F. gigantica*, while this enzyme excised the region in *F. hepatica* (29). *Fasciola* species were differentiable using AvaΠ and DraΠ enzymes that cut 28s DNA segment (25). In addition, different *Fasciola* species were identified using TsaΙ enzyme (30). Our results are in agree with above-mentioned studies and indicated six polymorphic sites in ITS1 region of *Fasciola* species.

Phylogenic and genotypic analysis showed that *F. hepatica* and *F. gigantica* comprised the two major *Fasciola* species isolated from infected livestock (cattle and sheep) in Zabol. The *F. hepatica* isolates were further placed in two sub categories of H1 and H2 genotypes, while *F. gigantica* isolates were mapped into four genotypic subcategories including G1, G2, G3, and G4. This high genetic variability may be related to the geographical location of Zabol City which is adjacent to Afghanistan and Pakistan countries from which livestock have entered the city by traditional routes.

Despite differences in multi-nucleotide sequences obtained from *Fasciola* isolates, the sequences showed 98%–100% homology with the sequences in GenBank.

From the limitation of present study, we can note unavailability of *Fasciola* isolates from livestock such as camel, goat, and a unique cow namely “Sistan cow” despite great efforts. In addition, we used a single gene to detect *Fasciola* species which may limit generalizability of our findings for intermediate species.

## Conclusion

All Seventy *Fasciola* samples from two different hosts (cattle and sheep) were identified as either *F. hepatica* or *F. gigantica* by PCR-RFLP. Comparison of *F. hepatica* and *F. gigantica* sequences obtained in our study showed nearly 100% homology with the sequences available at the GenBank, but sequences related to *F. hepatica* and *F. gigantica* showed few differences respective to each other. In addition, the isolates of *F. hepatica* belonged to H1 and H2 haplotypes, while those of *F. gigantica* belonged to G1, G2, G3 and G4 haplotype. No intermediate species were detected, however, this result is based on the evidence from single gen, and it needs to be validated by more investigations.

## Ethical considerations

Ethical issues (Including plagiarism, informed consent, misconduct, data fabrication and/or falsification, double publication and/or submission, redundancy, etc.) have been completely observed by the authors.
